# Global burden of children and adolescents' nutritional deficiencies from 1990 to 2021

**DOI:** 10.3389/fped.2025.1583167

**Published:** 2025-07-08

**Authors:** Juan Zhou, Yangmei Li, Yixi Cai

**Affiliations:** ^1^ Department of General Practice, Lijia Community Health Service Center of Chongqing Liangjiang New Area, Chongqing, China; ^2^ Outpatient Department, People’s Hospital of Chongqing Liangjiang New Area, Chongqing, China; ^3^ Department of Child Care, People’s Hospital of Chongqing Liangjiang New Area, Chongqing, China

**Keywords:** nutritional deficiencies, global burden of disease, children, adolescent, disability-adjusted life years, death

## Abstract

**Objective:**

Nutritional deficiencies critically impair growth and development in children and adolescents, yet comprehensive assessments of their global adolescent-specific burden are lacking. Adolescence represents a critical developmental window marked by rapid physical, cognitive, and psychosocial changes, making individuals particularly vulnerable to nutritional imbalances. This study aims to quantify the global burden of nutritional deficiencies in children and adolescents aged 0–19 years.

**Methods:**

Using 2021 Global Burden of Disease (GBD) data, we assessed the global impact of nutritional deficiencies among children and adolescents by analyzing rates and absolute numbers. Joinpoint analysis and average annual percentage changes (AAPC) were applied to explore temporal trends from 1990 to 2021.

**Results:**

In 2021, nutritional deficiencies caused 85,886 deaths [95% uncertainty interval (UI): 78,203–93,452] and 25.6 million DALYs (UI: 23.3–27.9 million) among children and adolescents globally. Global mortality and DALY rates due to nutritional deficiencies in this population declined consistently from 1990 to 2021. Notably, the DALYs rates for protein-energy malnutrition, iodine deficiency, vitamin A deficiency, and dietary iron deficiency decreased globally, with the AAPC from 1990 to 2021 being −5.2 (−6.4 to −4), −2.8 (−3 to −2.6), −2.6 (−2.7 to −2.6), and −0.5 (−0.6 to −0.5), respectively. Despite these improvements, regions with lower Social Development Index (SDI), including low and low-middle SDI areas, the death rates and DALYs rates for nutritional deficiencies among children and adolescents remain high, although they have been declining over the 30-year study period. Africa and Asia continue to bear the greatest burden. At the age level, children under five exhibited the highest burden across all age groups.

**Conclusions:**

Since 1990, the global burden of nutritional deficiencies among children and adolescents have declined; however, it continues to be a significant public health issue, particularly in regions with low SDI. To mitigate this burden, more effective public health interventions are required.

## 1 Introduction

Nutritional deficiencies refer to a disease characterized by significant deficits in growth and development among children and adolescents (individuals aged 0–19 years) due to insufficient nutrition, including but not limited to proteins, vitamins, minerals, and other essential nutrients (1, 2). Currently, nearly one-third of the world's population struggles with insufficient nutrition, presenting one of the most severe global community challenges (3). Previous research has consistently shown that nutritional deficiencies in childhood are linked to stunted growth, increased susceptibility to infectious diseases, and elevated risks of morbidity and mortality later in life (4, 5). In adolescence, a second phase of rapid physical and psychosocial development further increases nutritional demands, making this group particularly vulnerable to micronutrient imbalances and protein-energy malnutrition (6). Inadequate nutrition during adolescence can lead to delayed puberty, reduced peak bone mass, poor academic outcomes, and greater risk of chronic diseases in adulthood (6). Thus, addressing the burden of nutritional deficiencies in children and adolescents is critical for improving global health.

The Global Burden of Disease (GBD) database is currently the most comprehensive and reliable source of information on disease burden worldwide (7), with the latest update extending to 2021. Previous research has examined the incidence rates and disability-adjusted life years (DALYs) associated with nutritional deficiencies, including studies by Qing et al. (8) on the global burden from 1990 to 2019, and Pradhananga et al. (9) on protein-energy malnutrition in Nepal and India during the same period. Liu et al. (10) also assessed the burden of nutritional deficiencies among children under 15 years of age. However, despite these valuable contributions, there has been no comprehensive study investigating the global burden of nutritional deficiencies specifically among children and adolescents aged 0–19 years, based on the most recent available data.

To address this gap, this study aims to analyze mortality and DALY rates related to nutritional deficiencies in children and adolescents from 1990 to 2021, using the most up-to-date data from the GBD database. The study further explores how these rates correlate with national socioeconomic conditions, seeking to identify trends and provide insights that could inform the development of targeted interventions and healthcare policies aimed at alleviating nutritional deficiencies in children and adolescents. By stratifying the analysis across age groups—including 1–5 months, 6–11 months, 12–23 months, 2–4 years, 5–9 years, 10–14 years and 10–19 years—this study also provides critical context to understand how the burden evolves across developmental stages, and why both childhood and adolescence are essential periods for nutrition-focused interventions.

## 2 Materials and methods

### 2.1 Data sources

All data used in this study were obtained from the GBD 2021 database published by the Institute for Health Metrics and Evaluation (IHME) at the University of Washington. The GBD 2021 database provides disease burden data for 371 diseases and injuries across 204 countries and regions, along with disease burden data for 88 risk factors (11). All countries and territories were additionally classified into quintiles according to their SDI values: low SDI (<20th percentile), low-middle SDI (20–39th), middle SDI (40–59th), high-middle SDI (60–79th), and high SDI (≥80th) (12). Based on epidemiological similarity and geographical proximity, they were further divided into 23 GBD regions, including Andean Latin America, Australasia, and Southern Latin America (13). The indicators of disease burden include death cases, death rates, DALYs cases and DALYs rates. As the data provided by the GBD database are publicly accessible, informed consent from patients was not necessary for this study. In this study, we included children and adolescents under the age of 20 between 1990 and 2021. According to the definitions by the World Health Organization (WHO) and the United Nations Children's Fund (UNICEF), children were defined as those aged 0–17 years, while adolescents refer to individuals between 10 and 19 years old (14, 15). Following the GBD classification, this population was further divided into seven distinct age groups: 1–5 months, 6–11 months, 12–23 months, 2–4 years, 5–9 years, 10–14 years and 10–19 years.

### 2.2 Indicators analysis

The GBD 2021 database was accessed through the IHME website (http://ghdx.healthdata.org) to extract data on the global burden of nutritional deficiencies in children and adolescents from 1990 to 2021. In the “GBD Estimate” category, “cause of death or injury” was selected, with “Cause” set to “Nutritional deficiencies: Protein-energy malnutrition, Iodine deficiency, Vitamin A deficiency, and Dietary iron deficiency”. The primary indicators included the number and rate of deaths and DALYs. Each metric was calculated using 95% uncertainty intervals (UIs), determined from the 2.5th and 97.5th percentiles of 1000 samples drawn from the uncertainty distribution. Data were extracted for age groups under 19 years. DALYs represent the total years of healthy life lost due to disability and premature death, calculated as the sum of YLLs and YLDs (16).

### 2.3 Statistical analyses

Data were organized using Excel 2021, and the rate of change of death and DALYs was applied to assess trends in global children and adolescents' nutritional deficiencies burden indicators from 1990 to 2021. The rate of change was calculated as: (value in 2021—value in 1990)/value in 1990 × 100%. A joinpoint regression model (JRM) (17) was used to analyze trends in global children and adolescents' nutritional deficiencies deaths and DALYs rates from 1990 to 2021. Using Joinpoint, deaths and DALYs rates were log-transformed, and the optimal number of joinpoints was selected based on the curve-fitting performance recommended by the software. We fitted the model using a maximum of five joinpoints. To determine the magnitude and direction of trend changes, the average annual percentage change (AAPC) and 95% confidence interval (CI) were calculated for each type of nutritional deficiency. AAPC is calculated by geometrically weighting the annual percentage changes (APC) across segments according to segment length (18). Specifically, AAPC > 0 indicates an upward trend, AAPC = 0 indicates no significant change, and AAPC < 0 indicates a downward trend. If the 95% CI for AAPC includes 0, the trend is considered stable (19). Two-sided *P*-values for APC and AAPC were derived based on the two-sided *t*-test distribution, with a significance level of *α* = 0.05 (20). Jointpoint Regression Program 4.9.1.0 (National Cancer Institute, NCI, USA) and Excel 2021 (Microsoft Corporation, USA) were used in the joint regression analysis and table generation. R version 4.4.1 was used for statistical analyses (21–25).

## 3 Results

### 3.1 Trends in the global burden of deaths and DALYs attributable to nutritional deficiencies, 1990–2021

From 1990 to 2021, there was a substantial global decline in the number and rate of deaths and DALYs attributable to nutritional deficiencies among children and adolescents (aged 0–19 years). During this period, the number of children and adolescents' deaths due to nutritional deficiencies dropped markedly, indicating a substantial improvement in nutrition-related health outcomes among children and adolescents. The global death rate decreased by approximately 83%, and the DALY rate fell by about 63%, both of which reflect statistically significant reductions. Notably, males consistently exhibited lower death and DALY rates compared to females in both 1990 and 2021, suggesting a persistent sex disparity in nutritional vulnerability (Tables 1, 2).

**Table 1:** Death and rates of nutritional deficiencies among age under 19 years in 1990 and 2021.

Subgroups	1990		2021		Relative change in death rate, 1990–2021
	No. death cases (95% UI)	Death rates per 100,000 (95% UI)	No. death cases (95% UI)	Death rates per 100,000 (95% UI)	
Global	435,956.09 (360,601.86, 550,882.42)	19.3 (15.97, 24.39)	85,885.61 (66,545.59, 106,199.45)	3.26 (2.52, 4.03)	−0.83 (−0.87, −0.79)
Sex					
Male	205,165.64 (168,890.21, 258,323.63)	17.73 (14.59, 22.32)	42,978.41 (32,464.95, 54,283.5)	3.16 (2.39, 4)	−0.82 (−0.86, −0.76)
Female	230,790.45 (186,889.52, 289,336.5)	20.96 (16.97, 26.27)	42,907.2 (34,079.06, 52,083.11)	3.36 (2.67, 4.08)	−0.84 (−0.87, −0.79)
SDI					
Low SDI	177,179.74 (134,784.08, 236,859.13)	63.37 (48.21, 84.72)	55,419.55 (39,982.9, 71,273.74)	9.49 (6.84, 12.2)	−0.85 (−0.88, −0.8)
Low-middle SDI	183,251.07 (150,713.58, 224,308.06)	31.01 (25.5, 37.95)	22,404.45 (18,039.69, 27,302.27)	2.93 (2.36, 3.57)	−0.91 (−0.93, −0.87)
Middle SDI	65,890.48 (58,535.55, 75,162.55)	8.62 (7.66, 9.83)	7,356.92 (6,210.4, 8,660.52)	0.98 (0.83, 1.16)	−0.89 (−0.91, −0.86)
High-middle SDI	8,944.52 (7,818.13, 10,245.66)	2.42 (2.11, 2.77)	538.56 (453.4, 630.53)	0.18 (0.15, 0.21)	−0.93 (−0.94, −0.91)
High SDI	461.86 (368.27, 624.32)	0.18 (0.15, 0.25)	97.2 (87.65, 105.27)	0.04 (0.04, 0.05)	−0.77 (−0.83, −0.71)
Regions					
Southeast Asia, East Asia, and Oceania	46,165.67 (39,165.12, 56,985.82)	6.76 (5.73, 8.34)	3,563.57 (2,916.61, 4,272.85)	0.61 (0.5, 0.74)	−0.91 (−0.93, −0.88)
Central Europe, Eastern Europe, and Central Asia	893.92 (818.41, 975.97)	0.65 (0.59, 0.71)	126.09 (104.55, 153.34)	0.12 (0.1, 0.15)	−0.81 (−0.85, −0.77)
Latin America and Caribbean	27,801.39 (25,768.49, 30,056.83)	14.95 (13.86, 16.16)	3,447.02 (2,774.52, 4,343.14)	1.81 (1.45, 2.28)	−0.88 (−0.9, −0.85)
High-income	1,001.35 (942.48, 1,064.98)	0.39 (0.37, 0.42)	128.49 (115.13, 142.97)	0.05 (0.05, 0.06)	−0.86 (−0.88, −0.84)
World Bank Regions	435,726.15 (360,397.73, 550,605.98)	19.31 (15.98, 24.41)	85,816.53 (66,486.66, 106,113.56)	3.26 (2.52, 4.03)	−0.83 (−0.87, −0.79)
Sub-Saharan Africa	172,613.62 (131,049.91, 233,564.12)	62.58 (47.51, 84.67)	60,999.16 (44,054.28, 78,161.93)	10.15 (7.33, 13.01)	−0.84 (−0.87, −0.79)
North Africa and Middle East	11,974.53 (9,109.15, 17,868.78)	6.77 (5.15, 10.11)	2,501.57 (1,931.97, 3,206.39)	1.06 (0.82, 1.36)	−0.84 (−0.89, −0.78)
South Asia	175,505.62 (140,519.23, 214,954.04)	32.36 (25.91, 39.63)	15,119.71 (11,409.16, 19,892.24)	2.21 (1.67, 2.91)	−0.93 (−0.95, −0.9)
WHO region	435,672.15 (360,342.3, 550,550.21)	19.38 (16.03, 24.49)	85,809.36 (66,479.85, 106,105.99)	3.26 (2.53, 4.04)	−0.83 (−0.87, −0.79)
OECD Countries	9,784.67 (8,915.08, 10,749.36)	2.8 (2.55, 3.07)	1,129.92 (885.4, 1,449.73)	0.35 (0.28, 0.45)	−0.87 (−0.9, −0.84)
Sahel Region	50,344.01 (37,548.08, 69,262.57)	60.91 (45.43, 83.79)	25,515.9 (18,066.38, 33,666.17)	11.48 (8.13, 15.15)	−0.81 (−0.86, −0.75)
League of Arab States	14,385.4 (10,741.15, 20,121.78)	12.06 (9.01, 16.87)	5,112.85 (3,590.25, 7,163.81)	2.78 (1.95, 3.9)	−0.77 (−0.85, −0.69)
African Union	178,019.95 (136,595.35, 241,856.58)	51.23 (39.31, 69.6)	61,749.91 (44,613.97, 79,139.05)	8.77 (6.34, 11.24)	−0.83 (−0.87, −0.78)
Four World Regions	435,712.7 (360,384.45, 550,592.86)	19.32 (15.98, 24.41)	85,814.2 (66,484.29, 106,111.13)	3.26 (2.52, 4.03)	−0.83 (−0.87, −0.79)
European Union	71.91 (69.1, 74.85)	0.06 (0.06, 0.07)	29.29 (25.68, 33)	0.03 (0.03, 0.04)	−0.49 (−0.55, −0.42)
World Bank Income Levels	435,727.58 (360,399.15, 550,608.05)	19.31 (15.97, 24.4)	85,816.62 (66,486.73, 106,113.64)	3.26 (2.52, 4.03)	−0.83 (−0.87, −0.79)
G20	164,469.31 (134,769.08, 201,115.56)	11.52 (9.44, 14.08)	14,589.76 (11,269.41, 18,782.48)	1.07 (0.83, 1.38)	−0.91 (−0.93, −0.87)
Organization of Islamic Cooperation	142,213.5 (117,093.28, 176,669.18)	26.12 (21.5, 32.44)	37,942.09 (28,221.99, 48,129.1)	4.48 (3.33, 5.69)	−0.83 (−0.86, −0.78)
Commonwealth	233,767.22 (188,365.99, 290,095.58)	32.03 (25.81, 39.74)	36,786.89 (28,689.75, 45,471.56)	3.52 (2.75, 4.36)	−0.89 (−0.92, −0.85)
Health System Grouping Levels	435,727.66 (360,399.24, 550,608.12)	19.31 (15.97, 24.4)	85,816.68 (66,486.78, 106,113.68)	3.26 (2.52, 4.03)	−0.83 (−0.87, −0.79)
Gulf Cooperation Council	315.87 (210.34, 493.57)	2.88 (1.92, 4.5)	21.91 (17.83, 27.27)	0.14 (0.12, 0.18)	−0.95 (−0.97, −0.93)
Association of Southeast Asian Nations	19,896.59 (15,398.09, 27,081.66)	9.37 (7.25, 12.75)	2,693.2 (2,156.85, 3,277.83)	1.21 (0.97, 1.48)	−0.87 (−0.91, −0.82)
Nordic Region	0.95 (0.89, 1.03)	0.02 (0.02, 0.02)	0.53 (0.47, 0.6)	0.01 (0.01, 0.01)	−0.48 (−0.54, −0.4)

DALYs, disability-adjusted life years.

**Table 2:** DALYs number and rates of nutritional deficiencies among age under 19 years in 1990 and 2021.

Subgroups	1990		2021		Relative change in DALYs rate, 1990–2021
	No. DALYs cases (95% UI)	DALYs rates per 100000 (95% UI)	No. DALYs cases (95% UI)	DALYs rates per 100,000 (95% UI)	
Global	59,366,020.76 (49,645,061.49, 71,900,138.55)	2,628.47 (2,198.07, 3,183.43)	25,613,665.31 (19,356,140.15, 33,693,265.79)	971.75 (734.34, 1,278.27)	−0.63 (−0.69, −0.57)
Sex					
Male	28,365,426.08 (23,683,945.44, 34,539,944.39)	2,450.84 (2,046.35, 2,984.33)	11,543,315.57 (8,866,652.5, 15,023,341.5)	849.71 (652.68, 1,105.88)	−0.65 (−0.71, −0.59)
Female	31,000,594.68 (25,819,218.35, 37,750,326.81)	2,815.17 (2,344.65, 3,428.11)	14,070,349.74 (10,578,645.38, 18,850,852.68)	1,101.53 (828.17, 1,475.78)	−0.61 (−0.68, −0.54)
SDI					
Low SDI	20,284,679.33 (16,304,120.38, 25,910,950.47)	7,255.51 (5,831.72, 9,267.94)	11,583,359.52 (8,986,162.77, 14,815,811.35)	1,982.75 (1,538.18, 2,536.06)	−0.73 (−0.78, −0.67)
Low-middle SDI	25,170,708.23 (20,953,437.4, 30,405,703.09)	4,258.83 (3,545.28, 5,144.58)	9,142,515.49 (6,813,452.51, 12,404,243.94)	1,196.06 (891.36, 1,622.77)	−0.72 (−0.78, −0.66)
Middle SDI	11,319,440.1 (9,477,406.67, 14,034,652.17)	1,480.53 (1,239.6, 1,835.67)	4,047,042.76 (2,903,013.52, 5,593,921.89)	540.19 (387.49, 746.66)	−0.64 (−0.69, −0.58)
High-middle SDI	2,175,954.63 (1,722,916.65, 2,801,807.28)	587.83 (465.44, 756.91)	614,845.35 (419,523.32, 881,394.46)	202.68 (138.29, 290.54)	−0.66 (−0.71, −0.61)
High SDI	382,432.36 (266,940.1, 548,918.09)	152.17 (106.22, 218.41)	209,019.2 (143,232.11, 304,098.66)	89.81 (61.55, 130.67)	−0.41 (−0.47, −0.34)
Region					
Southeast Asia, East Asia, and Oceania	7,328,589.1 (6,081,329.18, 8,973,445.19)	1,072.36 (889.85, 1,313.04)	1,710,531.6 (1,233,062.02, 2,319,407.71)	294.61 (212.37, 399.48)	−0.73 (−0.78, −0.68)
Central Europe, Eastern Europe, and Central Asia	835,769.99 (589,309.89, 1,180,979.62)	605.09 (426.65, 855.02)	422,245 (286,958.76, 628,314.1)	404.68 (275.02, 602.17)	−0.33 (−0.4, −0.26)
Latin America and Caribbean	3,527,488.18 (3,132,190.64, 4,079,438.05)	1,897.03 (1,684.44, 2,193.86)	1,008,081.98 (751,468.92, 1,330,121.56)	528.32 (393.83, 697.1)	−0.72 (−0.77, −0.66)
High-income	316,857.61 (231,509.65, 442,143.07)	123.75 (90.41, 172.68)	174,147.22 (115,931.81, 259,087.84)	72.83 (48.48, 108.36)	−0.41 (−0.5, −0.31)
World Bank Regions	59,329,632.07 (49,614,237.05, 71,856,429.69)	2,629.94 (2,199.28, 3,185.22)	25,595,574.14 (19,342,617.55, 33,668,479.6)	971.95 (734.51, 1,278.51)	−0.63 (−0.69, −0.57)
Sub-Saharan Africa	18,779,809.09 (14,943,241.54, 24,344,274.04)	6,808.21 (5,417.35, 8,825.49)	11,191,976.09 (8,779,817.01, 14,221,687.76)	1,862.27 (1,460.9, 2,366.39)	−0.73 (−0.78, −0.67)
North Africa and Middle East	2,570,174.12 (1,989,390.8, 3,361,929.12)	1,453.95 (1,125.4, 1,901.84)	1,497,970.68 (1,082,687.43, 2,088,582.39)	633.41 (457.81, 883.15)	−0.56 (−0.64, −0.5)
South Asia	26,007,332.67 (21,297,560.11, 31,956,962.81)	4,794.61 (3,926.34, 5,891.46)	9,608,712.74 (6,923,366.94, 13,302,715.61)	1,405.82 (1,012.94, 1,946.28)	−0.71 (−0.77, −0.64)
WHO region	59,309,968.99 (49,597,942.2, 71,832,314.84)	2,638.55 (2,206.48, 3,195.63)	25,590,598.18 (19,339,280.83, 33,661,032.02)	973.38 (735.6, 1,280.35)	−0.63 (−0.69, −0.57)
OECD Countries	1,517,196.68 (1,282,976.77, 1,842,723.31)	433.53 (366.61, 526.55)	443,511.31 (324,545.44, 601,977)	138.1 (101.06, 187.45)	−0.68 (−0.73, −0.63)
Sahel Region	5,735,270.64 (4,553,901.04, 7,520,635.07)	6,938.45 (5,509.25, 9,098.36)	5,023,741.7 (3,886,054.94, 6,497,367.24)	2,261.23 (1,749.15, 2,924.53)	−0.67 (−0.75, −0.6)
League of Arab States	2,432,432.69 (1,927,321.59, 3,082,686.13)	2,039.5 (1,615.98, 2,584.71)	1,666,801.55 (1,226,321.45, 2,217,022.66)	906.43 (666.89, 1,205.65)	−0.56 (−0.65, −0.49)
African Union	19,859,595.25 (15,963,900.3, 25,692,634.7)	5,715.26 (4,594.15, 7,393.92)	11,736,881.37 (9,148,399.93, 14,993,235.51)	1,666.99 (1,299.35, 2,129.49)	−0.71 (−0.77, −0.64)
Four World Regions	59,326,327.27 (49,611,057.83, 71,853,519.88)	2,630.7 (2,199.9, 3,186.19)	25,594,742.21 (19,342,054.88, 33,667,305.23)	972.04 (734.57, 1,278.62)	−0.63 (−0.69, −0.57)
European Union	236,528.28 (159,474.77, 347,879.43)	209.6 (141.32, 308.28)	98,406.04 (64,504.9, 143,906.73)	109.16 (71.56, 159.64)	−0.48 (−0.54, −0.42)
World Bank Income Levels	59,333,160.48 (49,616,717.6, 71,861,609.77)	2,629.28 (2,198.71, 3,184.47)	25,596,761.14 (19,343,386.33, 33,670,111.27)	971.83 (734.41, 1,278.35)	−0.63 (−0.69, −0.57)
G20	26,642,232.42 (22,072,618.6, 33,051,223.29)	1,865.62 (1,545.64, 2,314.41)	9,043,775.41 (6,443,914.07, 12,623,213.95)	662.25 (471.87, 924.36)	−0.65 (−0.71, −0.58)
Organization of Islamic Cooperation	18,866,858.72 (15,887,126.24, 22,611,750.12)	3,464.58 (2,917.4, 4,152.26)	10,867,079.66 (8,223,382.94, 14,319,800.43)	1,284.15 (971.74, 1,692.15)	−0.63 (−0.7, −0.56)
Commonwealth	33,039,639.07 (27,198,901.4, 40,540,758.05)	4,526.39 (3,726.22, 5,554.04)	14,517,953.66 (10,809,505.98, 19,514,319.49)	1,390.96 (1,035.65, 1,869.65)	−0.69 (−0.75, −0.63)
Health System Grouping Levels	59,333,214.65 (49,616,760.07, 71,861,672.55)	2,629.27 (2,198.7, 3,184.46)	25,596,782.31 (19,343,401.45, 33,670,138.7)	971.83 (734.41, 1,278.35)	−0.63 (−0.69, −0.57)
Gulf Cooperation Council	109,187.71 (80,141.35, 148,523.46)	996.25 (731.23, 1,355.16)	47,422.01 (31,734.18, 69,901.09)	307.61 (205.85, 453.42)	−0.69 (−0.76, −0.62)
Association of Southeast Asian Nations	3,422,063.76 (2,770,159.18, 4,320,235.88)	1,611.28 (1,304.33, 2,034.18)	1,214,869.74 (875,459.46, 1,644,885.43)	547.04 (394.21, 740.67)	−0.66 (−0.73, −0.59)
Nordic Region	6,338.96 (3,857.84, 9,649.25)	108.52 (66.05, 165.2)	4,191.33 (2,526.59, 6,573.55)	67.56 (40.73, 105.96)	−0.38 (−0.5, −0.23)

DALYs, disability-adjusted life years.

Across all five SDI regions (low, low-middle, middle, high-middle, and high), both children and adolescents experienced significant reductions in death and DALY rates. The most substantial reductions were observed in high-middle and low-middle SDI regions, while high SDI regions exhibited a more modest but still significant decline. Despite overall improvements, notable disparities remained, with low-SDI regions continuing to bear a disproportionately high burden (Tables 1, 2).

At the GBD regional level, most areas saw a reduction of over 50% in death and DALY rates due to nutritional deficiencies among children and adolescents, with the exception of the Nordic Region, the European Union, and High-income regions, where the declines were less pronounced. The Sahel Region reported the highest death and DALY rates for this age group related to nutritional deficiencies in 2021, followed by Sub-Saharan Africa and the African Union, indicating that nutritional challenges remain especially severe in these regions (Tables 1, 2).

### 3.2 The average annual percent change of rate in deaths and DALYs

Joinpoint analysis indicated that from 1990 to 2021, global children and adolescents’ death rates due to nutritional deficiencies showed an overall fluctuating downward trend (AAPC = −5.6, 95% CI: −6.9 to −4.3, *p* < 0.001). Similarly, DALYs rates for nutritional deficiencies exhibited a fluctuating downward trend overall (AAPC = −3.2, 95% CI: −3.9 to −2.5, *p* < 0.001). The DALYs rate for protein-energy malnutrition also declined with fluctuations (AAPC = −5.2, 95% CI: −6.4 to −4.0, *p* < 0.001), as did the DALYs rate for iodine deficiency (AAPC = −2.8, 95% CI: −3.0 to −2.6, *p* < 0.001). The DALYs rate for vitamin A deficiency showed a decreasing trend (AAPC = −2.6, 95% CI: −2.7 to −2.6, *p* < 0.001), while the DALYs rate for dietary iron deficiency also declined (AAPC = −0.5, 95% CI: −0.6 to −0.5, *p* < 0.001) (Table 3).

**Table 3:** The average annual percent change of rate in deaths and DALYs.

Measure	Cause	AAPC	Lower CI	Upper CI	*p*-value
Deaths	Nutritional deficiencies	−5.6	−6.9	−4.3	<0.001
DALYs	Nutritional deficiencies	−3.2	−3.9	−2.5	<0.001
DALYs	Protein-energy malnutrition	−5.2	−6.4	−4	<0.001
DALYs	Iodine deficiency	−2.8	−3.0	−2.6	<0.001
DALYs	Vitamin A deficiency	−2.6	−2.7	−2.6	<0.001
DALYs	Dietary iron deficiency	−0.5	−0.6	−0.5	<0.001

AAPC, average annual percent change; DALYs, disability-adjusted life years.

**Figure 1:**
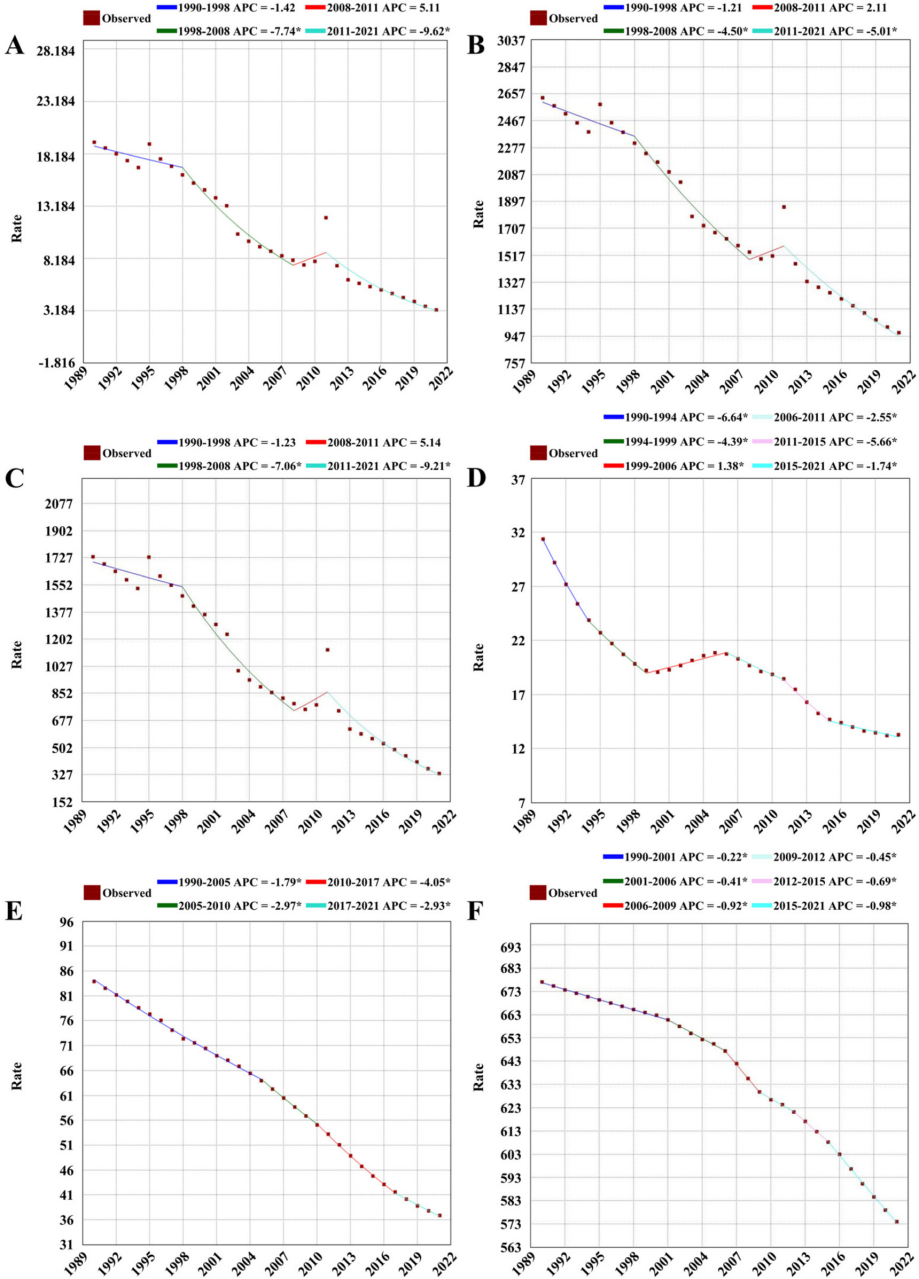
The joinpoint regression of global nutritional deficiencies during 1990 and 2021. **(A)** Denotes deaths rate of nutritional deficiencies; **(B)** Denotes DALYs rate of nutritional deficiencies; **(C)** Denotes DALYs rate of protein-energy malnutrition; **(D)** Denotes DALYs rate of iodine deficiency; **(E)** Denotes DALYs rate of Vitamin A deficiency; **(F)** Denotes DALYs rate of dietary iron deficiency.

Specifically, from 1990 to 1998, the global children and adolescents' death rate due to nutritional deficiencies began to decline slowly, followed by a rapid decrease from 1998 to 2008 (APC = −7.74, *p* < 0.05). A slight, nonsignificant increase occurred from 2008 to 2011 (APC = 5.11, *p* > 0.05), before a renewed rapid decline from 2011 to 2021 (APC = −9.62, *p* < 0.05) (Figure 1A). The global children and adolescents' DALYs rate for nutritional deficiencies showed a similar pattern: a slow decline from 1990 to 1998, followed by a rapid decrease from 1998 to 2008 (APC = −4.50, *p* < 0.05), a slight, nonsignificant increase from 2008 to 2011 (APC = 2.11, *p* > 0.05), and then a renewed rapid decline from 2011 to 2021 (APC = −5.01, *p* < 0.05) (Figure 1B). The DALYs rate for protein-energy malnutrition in children and adolescents showed a slow decline from 1990 to 1998, followed by a rapid decline from 1998 to 2008 (APC = −7.06, *p* < 0.05), a nonsignificant increase from 2008 to 2011 (APC = 5.14, *p* > 0.05), and a further rapid decline from 2011 to 2021 (APC = −9.21, *p* < 0.05) (Figure 1C). For iodine deficiency, the DALYs rate for children and adolescents declined sharply from 1990 to 1994 (APC = −6.64, *p* < 0.05), continued to decrease from 1994 to 1999 (APC = −4.39, *p* < 0.05), increased slightly from 1999 to 2006 (APC = 1.38, *p* < 0.05), and subsequently declined in three periods: 2006–2011 (APC = −2.55, *p* < 0.05), 2011–2015 (APC = −5.66, *p* < 0.05), and 2015–2021 (APC = −1.74, *p* < 0.05) (Figure 1D). The DALYs rate for vitamin A deficiency in children and adolescents declined at varying speeds across four periods: 1990–2005 (APC = −1.79, *p* < 0.05), 2005–2010 (APC = −2.97, *p* < 0.05), 2010–2017 (APC = −4.05, *p* < 0.05), and 2017–2021 (APC = −2.93, *p* < 0.05) (Figure 1E). For dietary iron deficiency, the DALYs rate for children and adolescents also showed a steady decline over six periods: 1990–2001 (APC = −0.22, *p* < 0.05), 2001–2006 (APC = −0.41, *p* < 0.05), 2006–2009 (APC = −0.92, *p* < 0.05), 2009–2012 (APC = −0.45, *p* < 0.05), 2012–2015 (APC = −0.69, *p* < 0.05), and 2015–2021 (APC = −0.98, *p* < 0.05) (Figure 1F).

### 3.3 The global burden of DALYs rates in nutritional deficiency across 204 countries

**Figure 2:**
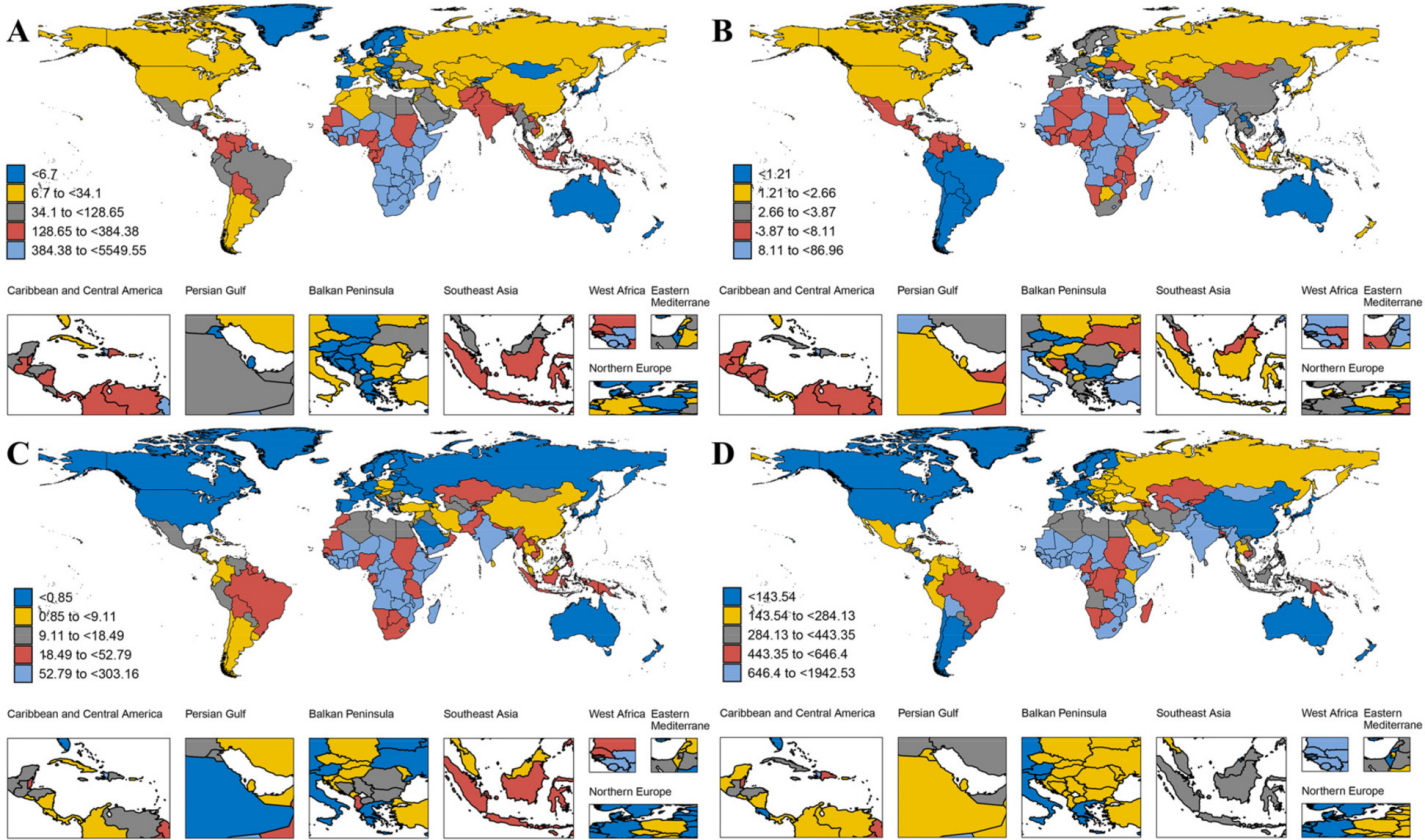
The global burden of DALYs rates in nutritional deficiency across 204 countries in 2021. **(A)** Denotes of protein-energy malnutrition; **(B)** denotes iodine deficiency; **(C)** denotes Vitamin A deficiency; **(D)** denotes dietary iron deficiency.

The global heatmap revealed substantial variation in the burden of nutritional deficiencies among children and adolescents across countries and regions. In 2021, Sierra Leone recorded the highest DALYs rate for protein-energy malnutrition among children and adolescent globally, at 5,549.55 per 100,000 (95% UI: 3,469.95–8,321.28). The highest DALYs rate for iodine deficiency among children and adolescents was observed in the Democratic Republic of the Congo, at 86.96 per 100,000 (95% UI: 44.54–154.17). Somalia had the highest DALYs rate for vitamin A deficiency in children and adolescents, at 303.16 per 100,000 (95% UI: 192.01–478.03). Yemen recorded the highest DALYs rate for dietary iron deficiency among children and adolescents, at 1,942.53 per 100,000 (95% UI: 1,335.09–2,696.42) (Figure 2).

### 3.4 Comparison of nutritional deficiency between men and women in different age groups

**Figure 3:**
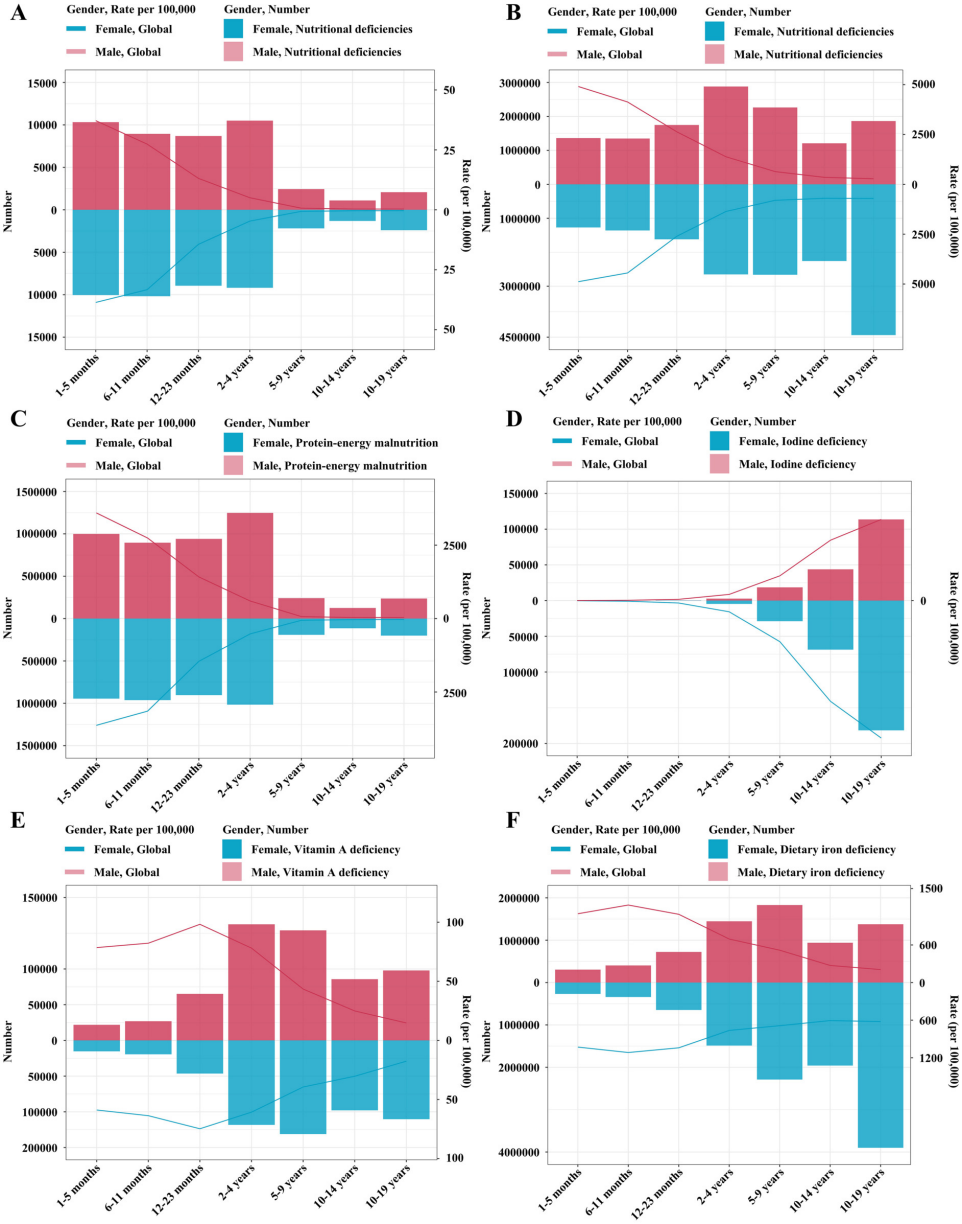
Comparison of nutritional deficiency between men and women in different age groups in 2021. **(A)** Denotes deaths number and rate of nutritional deficiencies. **(B)** Denotes DALYs number and rate of nutritional deficiencies. **(C)** Denotes DALYs number and rate of protein-energy malnutrition. **(D)** Denotes DALYs number and rate of iodine deficiency. **(E)** Denotes DALYs number and rate of Vitamin A deficiency. **(F)** Denotes DALYs number and rate of dietary iron deficiency.

In 2021, the burden of nutritional deficiencies among children and adolescents globally displayed notable trends across different age groups and sexes (Figure 3). In Plot A, the number and rate of deaths due to nutritional deficiencies fluctuated across age groups, with males and females showing similar overall trends. The death rate generally declined in older age groups.

Plot B demonstrated the DALYs related to nutritional deficiencies, showing an overall downward trend in rates with increasing age. However, the number of DALYs peaked in the 10–19 years age group, especially among females.

In Plot C, protein-energy malnutrition showed the highest DALYs numbers in younger age groups, with a noticeable decrease in DALYs rates as age increased. Females generally had lower DALYs compared to males in younger age groups.

Plot D illustrated DALYs due to iodine deficiency, with the lowest DALYs numbers in early life, followed by an increase in both number and rate with age. Females showed higher DALYs numbers in most age groups.

In Plot E, vitamin A deficiency DALYs exhibited the highest burden in the 2–4 years age group for males and the 5–9 years age group for females, with rates consistently decreasing across age groups over 2 years and older. Males had slightly higher DALYs rates than females, particularly in the 2–4 years age group.

Finally, Plot F depicted dietary iron deficiency DALYs, where rates and numbers remained relatively stable across different age groups, but females tended to show a higher burden compared to males. In addition, iron deficiency was the most common nutritional deficiency affecting malnourished children and adolescents of both genders globally. Overall, the trends in these plots indicated that the burden of nutritional deficiencies varied by type, age, and sex, with the early growth period representing a critical period for certain deficiencies.

## 4 Discussion

This study utilized GBD 2021 data to examine global trends in the burden of nutritional deficiencies among children and adolescents (aged 0–19 years) from 1990 to 2021. The findings show a marked decline in both mortality and DALY rates associated with children and adolescents' nutritional deficiencies. This trend may be linked to the implementation and progress of the Millennium Development Goals (26).

In terms of regional disparities, the burden of nutritional deficiencies among children and adolescent varied significantly based on SDI. Countries in low-SDI regions exhibited a disproportionately higher burden, with death rates and DALY rates much higher than in high-SDI regions. These differences are driven by varying levels of healthcare access, parental awareness, and socioeconomic conditions. High-SDI countries benefit from better healthcare systems, education, and nutrition (27), while low- and middle-income countries continue to struggle with limited resources, poor education, and insufficient healthcare infrastructure (28). Studies in Bangladesh (29), Pakistan (30), and Zimbabwe (31) consistently show that better socio-economic status reduces malnutrition. Strengthening interregional collaboration is essential to share medical advancements and health education, improving healthcare in resource-limited areas.

The global death and DALYs rates for children and adolescents' nutritional deficiencies, including protein-energy malnutrition, iodine deficiency, vitamin A deficiency, and iron deficiency, have shown a declining trend. This improvement is largely attributed to increased consumption of animal-based foods, greater dietary diversity, and enhanced access to essential micronutrients (32). Additionally, advancements in global economic conditions, healthcare quality, and food supplementation programs have further contributed to this progress (33). In particular, large-scale strategies—such as micronutrient supplementation (e.g., iron and folic acid tablets) (34, 35), food fortification (e.g., iodized salt and vitamin A-enriched cooking oil) (36), and school-based feeding programs (37)—have played a pivotal role in reducing micronutrient deficiencies among children and adolescents. However, children and adolescents in regions such as the Sahel and Sub-Saharan Africa continue to experience significant burdens of nutritional deficiencies, a finding consistent with the GBD 2019 studies (10, 25, 27, 32). These regional disparities may stem from various factors, including geographic challenges, cultural practices, economic limitations, and demographic pressures (25). In Africa, rapid population growth, underdeveloped agricultural and industrial sectors, and insufficient healthcare infrastructure exacerbate malnutrition and related diseases (38). To accelerate progress in these high-burden regions, more effective public health interventions are urgently needed. This includes expanding access to children and adolescent-targeted supplementation programs, integrating nutrition services into school health platforms, enhancing nutrition-sensitive agricultural strategies, and scaling up behavior change campaigns that promote healthy eating and hygiene practices (39).

At the age level, our study identified that children under five years old bore the heaviest burden of nutritional deficiencies, with death rates in this age group constituting a substantial share of overall death rates in 2021. Preschool-aged children are especially vulnerable to various forms of malnutrition, including stunting, severe wasting, and deficiencies in vitamin A and zinc, as well as inadequate breastfeeding practices (40). These factors contribute to nearly one-third of deaths in children under five (41). Particularly in low- and middle-income populations, early growth within the critical first 1000 days of life is essential for preventing long-term malnutrition (42). Weight gained during this period strongly correlates with adult BMI, highlighting the need for targeted interventions during early childhood to mitigate future health risks.

Although progress has been made in reducing the overall burden of childhood malnutrition, protein-energy malnutrition and iron deficiency remain the primary contributors to childhood DALYs. Among these, iron deficiency is the most prevalent nutritional disorder, aligning with prior studies (43). This condition can impair neurodevelopment, reduce physical capacity, and heighten the risk of infections in young children (44). Additionally, protein-energy malnutrition represents one of the most severe manifestations of malnutrition, depriving children of essential amino acids crucial for growth and development (45). This deficiency hampers cellular and physical growth, weakens immune function, and slows overall development (46).

Despite this early life vulnerability, adolescents also faced unique nutritional risks that warranted specific attention. Iron deficiency anemia was particularly common among adolescent girls, likely due to increased demands associated with rapid growth, menstruation, and gender-based disparities in dietary intake (47). Dietary iodine deficiency also showed a distinct age pattern, with a notable increase during adolescence, especially in females, reflecting heightened thyroid hormone requirements for pubertal development and cognitive maturation (48). These observations highlighted the need to extend micronutrient monitoring and targeted interventions beyond early childhood, with particular emphasis on the unmet needs of adolescent girls.

Moreover, while the burden of protein-energy malnutrition and vitamin A deficiency was greatest during early childhood (49), these conditions were not fully resolved in adolescence. Many older adolescents, especially in low-resource settings, continued to experience inadequate intake of essential nutrients, which could compromise physical growth, delay sexual maturation, impair immune competence, and hinder educational and occupational outcomes (50). These persistent gaps suggested that nutrition programs focusing exclusively on the first years of life may have overlooked the evolving demands of adolescents.

Regarding sex differences, our analysis revealed that girls experienced higher DALYs rates due to nutritional deficiencies compared to boys. This aligns with findings from earlier research, which indicated that females across various age groups are at greater risk of malnutrition (10). Studies in Bangladesh focusing on acute malnutrition further highlighted significant sex-based disparities, with girls more susceptible to severe malnutrition, resulting in increased mortality and higher DALYs rates (51). These findings underscore the importance of investigating the biological, social, and cultural factors driving these differences.

This study represents the first thorough analysis and investigation of the nutritional deficiency deaths, DALYs burden, and their changing trends worldwide among children and adolescents. A notable aspect of this research is its comparative analysis of children and adolescents' nutritional deficiency trends across different countries and regions, utilizing AAPC and Joinpoint to identify patterns over the past 30 years. This methodology offers valuable insights into the global landscape of nutritional deficiency among children and adolescents, serving as a useful reference for future studies. This study had several limitations to consider. First, while GBD 2021 applied adjustments to account for data biases, low-quality sampling, survey methods, and methodological gaps, the reliability of findings on nutritional deficiency remains contingent on the quality and availability of data input into the models (21–23). In regions with limited health data infrastructure, particularly low- and middle-income areas, data may be incomplete (24). Second, the absence of a standardized diagnostic criterion and the interchangeable use of related terms may lead to underestimations of the prevalence and impact of nutritional deficiencies. Third, GBD 2021 focused on a limited set of deficiencies—namely, protein-energy malnutrition, dietary iron deficiency, vitamin A deficiency, and iodine deficiency—without sufficient data to comprehensively assess other deficiencies, such as vitamin C and folate (25). Notably, some key data, including dietary iron deficiency incidence, were excluded from the GBD 2021 study. Future research should incorporate additional metrics, such as the Health Assessment Questionnaire (HAQ), or an integrated set of databases, to improve precision in estimating disease burden and evaluating health outcomes. Finally, further investigation is required to understand the reasons behind the observed decrease in DALYs and the ongoing rise in nutritional deficiency incidence. Because of the reporting time lag in health data collection and its integration into GBD estimates, recent changes in health status might not yet be reflected in current estimates.

## 5 Conclusions

Since the 1990s, global efforts and public health initiatives have led to a substantial decline in nutritional deficiencies among children and adolescents, as reflected by significant reductions in both mortality and DALY rates. However, this downward trend has not been uniformed across regions. Children and adolescents in Asia and Africa, particularly those in low-SDI countries, continue to experience a disproportionately high burden. Nutritional deficiency in adolescence thus remains a pressing public health concern in these areas. Addressing this challenge requires that developing countries prioritize the strengthening of primary healthcare systems and adopt comprehensive, context-specific interventions. These should include improvements in early childhood education, enhanced nutritional support programs, and timely, proactive treatment strategies to mitigate long-term health consequences.

## Data Availability

The original contributions presented in the study are included in the article/Supplementary Material, further inquiries can be directed to the corresponding author.
